# Isolation and characterization of thermophilic *Campylobacter* species from geese raised in Kars region (Turkey) using cultural, molecular and mass spectrometry methods

**DOI:** 10.22099/IJVR.2021.41103.5962

**Published:** 2022

**Authors:** E. G. Demiroğlu, M. Şahin, F. Büyük

**Affiliations:** 1 Ph.D. Student in Microbiology, Department of Microbiology, Institute of Health Sciences, Kafkas University, Kars, 36100, Turkey;; 2 Department of Microbiology, Faculty of Veterinary Medicine, Kafkas University, Kars, 36100, Turkey

**Keywords:** Antibiotic susceptibility, Goose, MALDI-TOF MS, PCR, Thermophilic Campylobacter

## Abstract

**Background:**

Thermophilic *Campylobacters* are found in the digestive tract of wild and domestic poultry and can be transmitted to humans following their fecal discharges.

**Aims:**

This study aimed to isolate thermophilic *Campylobacter* by culture from cloacal swabs of geese, commonly breeding in Kars region, and to identify the isolates by PCR and mass spectrometry. Antibiotics susceptibility and resistance genes of the isolates were also analysed.

**Methods:**

The study included 400 cloacal swab samples of clinically healthy geese. The samples were cultured on mCCDA medium following the pre-enrichment in Preston broth. Identification of the isolates was performed by phenotypic methods, PCR, and MALDI-TOF MS. Antibiotic susceptibility and resistance genes of the isolates were analysed with the disc diffusion method and PCR, respectively.

**Results:**

Thermophilic *Campylobacter* spp. were isolated from 157 (39.3%) samples. 151 (96.2%) isolates were identified *Campylobacter jejuni* and 6 (3.8%) *Campylobacter coli* by the phenotypic tests and PCR. Among 125 isolates analysed by MALDI-TOF MS, 119 (95.2%) were identified *C. jejuni* and 6 (4.8%) *C. coli*. The isolates’ resistance to ampicillin, tetracycline, ciprofloxacin, gentamicin, and azithromycin were found 33.8%, 41.4%, 75.2%, 12.1%, and 7.6%, respectively. The distributions of *bla*_OXA61_, *tetO*, *gyrA*, and *aphA-3* genes were 3.2%, 90.8%, 50.8%, and 52.7%, respectively.

**Conclusion:**

Since geese are raised in pastures in the Kars region, protecting and not polluting the existing natural environment and preventing their contact with wild birds will prevent the spread of these microorganisms*.*

## Introduction

 In Turkey, goose breeding is intensively carried out in Kars and its surroundings. It has traditional consumption habits, especially dried goose meat. Geese have some known bacterial and viral diseases. It is important to determine the presence and frequency of *Campylobacter*, an important bacterial species in terms of human and animal health and carried by poultry in the digestive system. *Campylobacter* species are important in transmission to domestic and wild animals and humans by contaminating environments through their primary hosts (Jones, 2011). The transmission to humans usually occurs by the oral route through contact with contaminated poultry products. Cloacal colonization, leading to contamination of poultry meat, is an important factor in the epidemiology and transmission of the thermophilic species (Coker et al., 2002).

 Cloacal colonization of the thermophilic *Campylobacter* may vary with age and seasonality (Ringoir et al., 2002). In poultry, contamination and first colonization following fecal scattering begin at 2-3 weeks of age, and the spread of the microorganism to the flock increases with age (Evans and Sayers, 2000). The bacterial transmission among animals after the initial infection is extremely fast, and most of the animals in the herd are colonized in just a few days (Shreeve et al., 2000). While colonization continues for life in poultry, the persistence of colonization may vary among the *Campylobacter* species (Ringoir et al., 2002). It has also been reported that seasons are also effective in cloacal colonization (Refregier-Petton et al., 2001). Generally, a higher prevalence was reported in summer months when environmental exposure is increased (Evans, 1997).

 Isolation of the bacteria using cultural methods with mCCDA, Columbia, Skirrow, and Butzler agar is considered the gold standard in the diagnosis of thermophilic *Campylobacter*. Morphological, cultural, and biochemical tests are widely used to characterize the bacteria. However, alternative diagnostic methods are required due to the difficulties in the cultures, time-consuming process, or the morphological variations of *Campylobacter* in subcultures (live but non-culturable forms-VBNC). In this scope, PCR, which provides enzymatic amplification of the bacteria-specific genes, and MALDI-TOF MS, which provides protein profiles following the ionization of bacterial biomolecules and organic molecules, provide faster and more reliable results (Seng et al., 2009).

 No antibiotic treatment is usually required for *Campylobacter* infections in poultry, while it may be necessary in humans, especially in cases with severe symptoms or immunodeficiency. Macrolides such as erythromycin and azithromycin are the most commonly used antimicrobials. Fluoroquinolones, tetracyclines, and aminoglycosides are other alternative medicines (Hasdemir, 2007). However, as a result of the unconscious and widespread use of antibiotics as prophylactic and growth factors especially in poultry breeding, antibiotic resistance in thermophilic species becomes more common and even multi-drug resistances have emerged (Wieczorek and Osek, 2013). These resistances are mainly based-on the presence of some genes such as *aphA-1*, *aphA-3*, *aacA*, *aphD*, and *aac* for aminoglycosides (Lambert et al., 1985), *tetO* for tetracyclines (Chopra and Roberts, 2001), *gyrA* for fluoroquinolones (Luangtongkum et al., 2009), *ermB* for macrolides (Qin et al., 2014), and *bla*_OXA-61_ for β-lactams (Komba et al., 2015).

 This study aimed to isolate thermophilic *Campylobacter* from cloacal swab samples from geese of different age groups, which are widely raised in the Kars region, and to identify the isolates with molecular and MALDI-TOF MS. Another goal of the study is to determine the susceptibility of isolates to various antibiotics and to analyze the common resistance genes by PCR.

## Materials and Methods

**Study material**

 Ethical permission of the study was approved by the decision of Kafkas University Animal Experiments Local Ethics Committee, with the number “2019-124”.

 In this study, 400 cloacal swab samples were taken from clinically healthy geese from family-type farms in the center and districts of Kars (Turkey) between January and October 2020. In order to evaluate the seasonal distribution of thermophilic *Campylobacter*, sampling was done every month of the year with 40 cloacal samples.

**Isolation and identification of thermophilic** ***Campylobacter***

 The cloacal swab samples were pre-enriched in 5 ml *Brucella* broth with the addition of *Campylobacte*r growth supplement and Preston *Campylobacter* selective supplement under microaerobic conditions at 42°C for 48-72 h (Anonymous 1, 2020). Then, 20 µL pre-enriched content was inoculated onto mCCDA selective medium supplemented with *Campylobacter* Selective Supplement; the seeded agar plates were incubated under the same conditions. Following the incubation, colonies with 0.5 mm diameter, grayish color, sometimes metallic highlights, smooth surface but tendency to spread were evaluated as suspicious for thermophilic *Campylobacter* on mCCDA medium. Within the scope of preliminary identification of thermophilic *Campylobacter*, their microscopic morphology (Gram-negative, curved or comma-shaped bacillus), biochemical activities (oxidase and catalase activities, hippurate hydrolysis and indoxyl acetate reaction), and susceptibility to various antibiotics (nalidic acid and cephalothin) were evaluated (Anonymous 1, 2020).

**Molecular identification**

 DNA extraction from suspected colonies was performed with a single colony lysis buffer solution (SCLB) supplemented with heat treatment (Mamur, 1961).

 For the molecular identification of the thermophilic *Campylobacter* species, a multiplex polymerase chain reaction (mPCR) method was applied (Wang et al., 2002).

 For this purpose, total mPCR volume for each sample was adjusted as 25 µL with the components of 2.5 µL template DNA, 2.5 µL 10x PCR buffer, 3 µL MgCl_2_ (20 mM), 0.5 µL dNTP mix (0.2 mM), 1 µL of each primer (20 pmol) and 0.5 µL Taq polymerase (1.25 U). Thermal cycle of the mPCR was carried out by initial denaturation at 95°C for 6 min, followed by 30 cycles, with denaturation at 95°C for 30 s, primer attachment at 59°C for 30 s and elongation at 72°C for 30 s, and finally elongation at 72°C for 7 min. The amplified products were analyzed by 1.5% agarose gel electrophoresis, and products of 323 bp for *C. jejuni*, 126 bp for *C. coli*, 251 bp for *C. lari* and 204 bp for *C. upsaliensis* were evaluated (Table 1).

**Table 1:** Primer sequences used for mPCR

Target gene	Primer sequence (5´-3´)	Band size	Reference
*C. jejuni* (*hipO*)	ACTTCTTTATTGCTTGCTGC	323 bp	
	GCCACAACAAGTAAAGAAGC		
*C. coli* (*glyA*)	GTAAAACCAAAGCTTATCGTG	126 bp	
	TCCAGCAATGTGTGCAATG		
*C. lari* (*glyA*)	TAGAGAGATAGCAAAAGAGA	251 bp	
	TACACATAATAATCCCACCC		
*C. upsaliensis* (*glyA*)	AATTGAAACTCTTGCTATCC	204 bp	
	TCATACATTTTACCCGAGCT		

**Identification with mass spectrometry**

 Identification of the thermophilic *Campylobacter* species by MALDI-TOF MS (Vitek MS, Biomerıux) was performed in Malatya İnönü University (Turkey). The isolates were subjected to the routine culture processes in proper media, and a fresh colony was taken with a loop to place on the MALDI TOF plate. Then, 1 µL α-cyano-4-hydroxynamic acid (α-CHCA) matrix solution was added to the well and dried. Identification was done by leaving the plate in the MALDI-TOF MS device for analysis (Yakupoğulları et al., 2019).

**Determination of antibiotic susceptibility of the isolates**

 The susceptibility of the identified thermophilic *Campylobacter* species against azithromycin (15 µg), tetracycline (30 µg), ciprofloxacin (15 µg), gentamicin (10 µg), and ampicillin (10 µg) (OXOID) was determined by Kirby-Bauer disc diffusion method (Taremi et al., 2006). The inhibition zone diameters formed at the end of the test were measured and evaluated according to the CLSI 2017 criteria (CLSI, 2017). During the test, *Escherichia coli* ATCC^®^ 25922, *Escherichia coli* ATCC^®^ 35218, and *Pseudomonas aeruginosa* ATCC^®^ 27853 were used as control bacteria.

**Resistance gene analysis of the isolates**

 Of the phenotypically resistant thermophilic *Campylobacter* isolates, the resistance genes of tetracycline (*tetO*), gentamicin (*aphA-3*), ampicillin (*blaOXA-61*), azithromycin (*ermB*), and ciprofloxacin (*gyrA*) were analysed by PCR (Table 2).

**Statistical analysis**

 SPSS (Statistical Package for Social Sciences for Windows) 20.0 package software was used for statistical analysis. Mann-Whitney test was used for statistical comparison of the proportional distribution between two data values. The Kruskal-Wallis test was also employed for statistical comparisons of the proportional distribution between data of three or more experiments.

## Results

**Isolation and identification**

 As a result of cultural analysis, thermophilic *Campylobacter* isolation was performed in 157 (39.3%) of 400 goose cloacal swab samples. Out of 157 thermophilic *Campylobacter* isolates, 151 (96.2%) were identified as *C. jejuni* and 6 (3.8%) as *C. coli*. *C. lari* and *C. upsaliensis* were not detected in the study. Considering the cloacal colonization of thermophilic species by seasons, it was determined that the isolation rate started to increase in the spring, reached a peak in the summer, and gradually decreased in the autumn and winter (P<0.05) (Table 3).

 In order to test the age-related colonization of thermophilic C*ampylobacter*, ten 1-day-old goslings

**Table 2:** Primer sequences used for PCR to analyze resistance gene

Target gene	Primer sequence (5´-3´)	PCR reaction		
		Annealing (°C)	Band size	Reference
*tet(O)*	GGCGTTTTGTTTATGTGCG	51	559 bp	Pratt and Korolik (2005)
	ATGGACAACCCGACAGAAGC			
*aphA-3*	TGCGTAAAAGATACGGAAG	52	701 bp	Obeng *et al*. (2012)
	CAATCAGGCTTGATCCCC			
*bla*_OXA-61_	AGAGTATAATACAAGCG	46	372 bp	Obeng *et al*. (2012)
	TAGTGAGTTGTCAAGCC			
*ermB*	CAGGTAAAGGGCATTAACGACG	60	738 bp	Zhou *et al*. (2016)
	CATCTGTGGTATGGCGGGTAAG			
*gyrA*	GCTCTTGTTTTAGCTTGATGC	56	620 bp	
	TTGTCGCCATCCTACAGCTA			

**Table 3:** Sample characteristics and test results used in identification

Month	Sample size	Isolation rate	PCR	MALDI-TOF MS	Percentage (%)	Mann-Whitney P-value
January	40	11	10 *C. jejuni*, 1 *C. coli*	9 *C. jejuni*, 1 *C. coli*	27.5	P<0.05
February	40	13	12 *C. jejuni*, 1 *C. coli*	8 *C. jeuni*, 1 *C. coli*	32.5	
March	40	12	12 *C. jejuni*, -	8 *C. jejuni*, *-*	30	
April	40	15	14 *C. jejuni*, 1 *C. coli*	11 *C. jejuni*, 1 *C. coli*	37.5	
May	40	20	19 *C. jejuni*, 1 *C. coli*	17 *C. jejuni*, 1 *C. coli*	50	
June	40	21	20 *C. jejuni*, 1 *C. coli*	17 *C. jejuni*, 1 *C. coli*	52.5	
July	40	26	25 *C. jejuni*, 1 *C. coli*	19 *C. jejuni*, 1 *C. coli*	65	
August	40	23	23 *C. jejuni*, *-*	18 *C. jejuni*, *-*	57.5	
September	40	10	10 *C. jejuni*, *-*	6 *C. jejuni*, *-*	25	
October	40	6	6 *C. jejuni*, *-*	6 *C. jejuni*, *-*	15	
Total	400	157	151 *C. jejuni*, 6 *C. coli*	119 *C. jejuni*, 6 *C. coli*	39.3	

were sampled from the same farm, which was subjected to the same feeding and care conditions. While the agent could not be detected on the seventh day in the swab samples taken at regular intervals starting from the first week of May, a significant increase was found in colonization in parallel with the increasing age (P<0.05) (Fig. 1).

**Fig. 1:**
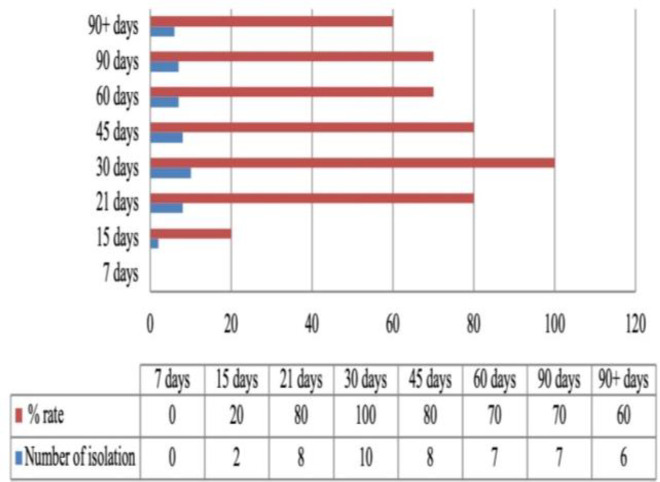
Daily age-related cloacal colonization of the thermophilic *Campylobacter*

**Molecular identification**

**Fig. 2:**
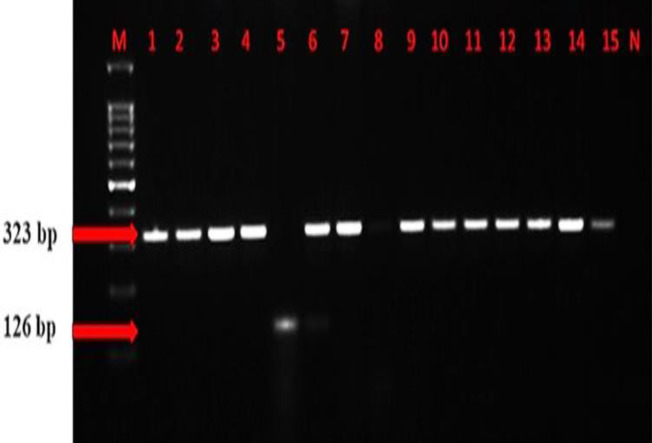
Electrophoresis of the mPCR analyses of the thermophilic *Campylobacter* species. M: DNA ladder (Hyperladder 100 bp plus/Bioline), and N: Negative control. Lanes 1-15: Thermophilic* Campylobacter* field isolates. Lane 5 shows a *C. coli* isolate with 126 bp and the others show *C. jejuni* isolates with 323 bp

 As a result of mPCR, 151 (96.2%) thermophilic *Campylobacter* isolates were identified as *C. jejuni* and 6 (3.8%) as *C. coli*, which was 100% consistent with the identification findings by the phenotypical methods (Fig. 2).

**Mass spectrometry**

 MALDI-TOF MS analysis was performed on only 125 isolates that could be sub-cultured. As a result of the analysis, 119 (95.2%) of the isolates were identified as *C. jejuni* and 6 (4.8%) as *C. coli* with a confidence interval of 99.9% (Appendix 1). MALDI-TOF MS identification findings were 100% compatible with the results of cultural analysis and mPCR (Table 3).

**Antibiotic susceptibility test**

 Antibiotic susceptibility test of 157 isolates, including 151 *C. jejuni* and 6 *C. coli*, was performed with ampicillin, tetracycline, ciprofloxacin, gentamicin, and azithromycin. In total, resistance to different antibiotics was determined in 144 isolates. The highest resistance was determined to ciprofloxacin with 118 isolates (116 *C. jejuni* and 2 *C. coli*). Tetracycline resistance was determined in 65 isolates (63 *C. jejuni* and

2 *C. coli*), ampicillin resistance in 53 (50 *C. jejuni* and 3 *C. coli*), gentamicin resistance in 19 (*C. jejuni*), and azithromycin resistance in 12 isolates (*C. jejuni*). *C. jejuni* isolates were found mostly resistant to ciprofloxacin (76.8%), while azithromycin was the more effective antibiotic. *C. coli* isolates were found mostly (50%) resistant to ampicillin, while 100% sensitivity was determined against gentamicin and azithromycin (Table 4).

**Resistance gene analysis**

**Fig. 3:**
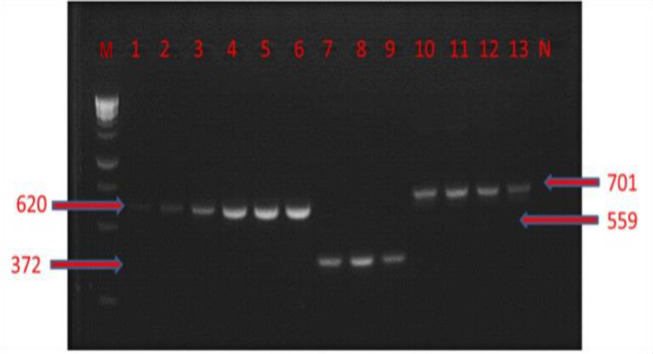
Gel electrophoresis image of PCR analysis of resistance genes. M: DNA ladder (HyperLadder 1 kb plus/Bioline), and N: Negative control. Lanes 1-3: *gyrA* gene with 620 bp, Lanes 4-6: *tetO* gene with 559 bp, Lanes 7-9: *blaOXA-61* gene with 372 bp, and Lanes 10-13: *aphA-3* gene with 701 bp

 The resistance gene ratios of thermophilic *Campylobacter* isolates are shown in Table 5 using phenotypic and molecular (PCR) assays. Among the thermophilic *Campylobacter* species, the most common resistance gene was *tetO* with 90.8% frequency, followed by *aphA-3* with 52.7%, *gyrA* with 51.7%, and *blaOXA-61* with 35.2% (Fig. 3). The isolates were negative for the *ermB* gene.

**Multiple drug resistance patterns**

 In the study, isolates with resistance to two or more antibiotics were evaluated as “multi-drug resistance (MDR)”. Among the thermophilic *Campylobacter* isolates total MDR rate was found as 52.87%. Double MDR was observed in 56 isolates (53 *C. jejuni* and 3 *C. coli*) such as TET-AMP in 5 (4 *C. jejuni* and 1 *C. coli*), CIP-AZM in 3 *C. jejuni*, GEN-AMP in 1 *C. jejuni*, CIP- AMP in 18 (16 *C. jejuni* and 2 *C. coli*), CIP-GEN in 2 *C. jejuni*, and TET-CIP in 27 *C. jejuni* isolates. Triple

**Table 4:** Antibiotic susceptibility profiles of the thermophilic *Campylobacter* isolates

Antibiotic	*C. jejuni*		Kruskal-Wallis P-value	*C. coli*		Kruskal-Wallis P-value	Total		Kruskal-Wallis P-value
	n:151	%		n:6	%		n:157	%	
Ampicillin	50	3.1	P<0.05	3	50	P<0.05	53	33.8	P<0.05
Tetracycline	63	41.7		2	33.3		65	41.4	
Ciprofloxacin	116	76.8		2	33.3		118	75.2	
Gentamicin	19	12.6		-	-	-	19	12.1	
Azithromycin	12	7.9		-	-	-	12	7.6	

**Table 5:** Resistance gene profiles of the thermophilic *Campylobacter* isolates obtained by PCR

Gene	Phenotypically resistant isolates	PCR positivity of resistance gene		Phenotypically resistant *C. jejuni*	PCR positivity of resistance gene of *C. jejuni* isolates			Phenotypically resistant *C. coli*	PCR positive of resistance gene of *C. coli* isolates		
		n (%)	Kruskal-Wallis P-value		n	%	Kruskal-Wallis P-value		n	%	Kruskal-Wallis P-value
*bla*_OXA-61_	53	19 (35.2)	P<0.05	50	17	34	P<0.05	3	2	66	-
*tetO*	65	59 (90.8)	P<0.05	63	58	98.3	P<0.05	2	1	50	-
*gyrA*	118	60 (51.7)	P<0.05	116	59	50.8	P<0.05	2	1	50	-
*aphA-3*	19	10 (52.7)	P<0.05	19	10	52.7	P<0.05	-	-	-	-
*ermB*	12	-	-	12	-	-	-	-	-	-	-

**Table 6:** Multi-drug resistance (MDR) profiles of the thermophilic *Campylobacter* isolates

MDR profile	*C. jejuni*	*C. coli*	Total
TET&AMP	4	1	5
CIP&AZM	3	-	3
GEN&AMP	1	-	1
CIP&AMP	16	2	18
CIP&GEN	2	-	2
TET&CIP	27	-	27
CIP&AMP&AZM	1	-	1
CIP&GEN&AMP	2	-	2
TET&CIP&AMP	10	-	10
TET&GEN&AMP	1	-	1
TET&CIP&GEN	5	-	5
TET&CIP&GEN&AZM	1	-	1
TET&CIP&GEN&AMP	4	-	4
TET&CIP&AMP&AZM	2	-	2
TET&CIP&AZM&AMP&GEN	1	-	1
Total	80	3	83

MDR was observed in 19 *C. jejuni* isolates such as CIP-AMP-AZM in 1, CIP-GEN-AMP in 2, TET-CIP-AMP in 10, TET-GEN-AMP in 1, and TET-CIP-GEN in 5 isolates. Quadruple MDR was observed in 7 *C. jejuni* isolates such as TET-CIP-GEN-AZM in 1, TET-CIP-GEN-AMP in 4, and TET-CIP-AMP-AZM in 2 isolates. Only one quintette MDR was observed in one *C. jejuni* and this isolate was almost assumed as “superbug” (Table 6).

## Discussion

 Cloacal carriage in poultry and subsequent scattering with faeces is an important epidemiological feature of the thermophilic *Campylobacter* for both human and animal infections. In studies, the prevalence of the thermophilic *Campylobacter* in cloacal swab or fecal samples of geese varies between 35% and 89.6% (Aydin et al., 2001; Elmalı et al., 2004; Colles et al., 2008; Moriarty et al., 2011). The prevalence of the thermophilic *Campylobacter* of the current study was found to be 39.3% in goose and showed a big harmony with the others (Aydin et al., 2001; Colles et al., 2008; Moriarty et al., 2011). However, the main reasons for the difference (Elmalı et al., 2004) can be caused by factors such as geographical, climate, and seasonal differences, cultivation style, and age ranges of sampled animals.

 Intestinal colonization of the thermophilic *Campylobacter* in poultry increases in parallel with the age and is lower in birds younger than 2-3 weeks due to the presence of maternal antibodies (Sahin et al., 2003) and natural gut flora (van der Wielen et al., 2000). This hypothesis is also confirmed by experimental studies (Newell, 2001; Boyd *et al*., 2005). In this study, while the thermophilic *Campylobacter* were not found in 7-day-old goslings, the cloacal colonization started on day 15 and reached 100% at day 30. These results were compatible with the results of studies indicating persistent negativity of the cloacal colonization until at least 10 days of age that was the so-called lag phase (Newell and Fearnley, 2003; Sahin et al., 2003). Similarly, a biased increase occurs in cloacal colonization towards the warmer months (May to October) in poultry, and this leads a similar seasonal course in human infections (Leflon-Guibout and Munier, 2016; Kalupahana et al., 2018; Es-Soucratti et al., 2020). The highest colonization rate was found in summer (58.3%) followed by spring (39.2%) months in the present study. This is in parallel with reports suggesting that increased cloacal colonization is likely due to several reasons, such as increased frequency of environmental exposure of birds and free access to water resources (Jorgensen et al., 2011; Leflon-Guibout and Munier, 2016).

 PCR in precise diagnosis and differentiation of the thermophilic *Campylobacter* species has been widely used. In this study, the compatibility of the PCR with the phenotypical methods in species differentiation was found to be similar to the reported studies. In addition, the dominancy (96.2%) of *C. jejuni* is also evident (Szosland-Faltyn et al., 2008; Wysok et al., 2020). Compared to the other methods, MALDI-TOF mass spectrometry is more advantageous in terms of speed and cost and enables a novel diagnostic approach (Seng et al., 2009). MALDI-TOF MS has also been used to identify *Campylobacter* species and has demonstrated high compatibility with PCR (Scerbova and Laukova, 2016; Nowaczek et al., 2019). In this study, MALDI-TOF MS analysis was performed for 125 isolates due to their high fragility in subcultures in which 119 (95.2%) were identified as *C. jejuni* and 6 (4.8%) as *C. coli* with 100% agreement with other tests. This high diagnostic agreement among identification methods is similar to that reported for the thermophilic *Campylobacter* species (Scerbova and Laukova, 2016; Nowaczek et al., 2019).

 The benign course of *Campylobacter* infections depends on applications both rapid diagnosis of the agent and accurate treatment of the infection. Birds do not require treatment because of their asymptomatic or mild clinical course. However, knowing the etiological and antibiotic susceptibility characteristics of the causative agent circulated in poultry can offer an insight into human beings. Macrolides, aminoglycosides, tetracyclines, fluoroquinolones, and beta-lactams are widely used in treatment of the thermophilic Campylobacters infections (Allos, 2001). Bacterial resistance to antibiotics is mostly caused by the genes encoded by plasmid or chromosomal DNA and their prevalence varies according to determinants such as host and environmental conditions. In this study, the rates of resistance and the prevalence of the common resistance gene for these antibiotics were either within the previously reported ranges (Aydin et al., 2001; Griggs et al., 2009; Bardoň et al., 2017; Szczepanska et al., 2017; Marotta et al., 2020; Proiettia et al., 2020; Wysok et al., 2020) or very close (Aydin et al., 2001; Bakeli et al., 2008; Çokal et al., 2009; Jamali et al., 2013; Abdi-Hachesoo et al., 2014; Bardoň et al., 2017; Bailey et al., 2019; Melo et al., 2019; Silva et al., 2019). Indiscriminate use of antibiotics in the treatment of clinically important bacterial infections causes multidrug resistance. Multiple resistance profiles have been reported against many antimicrobial agents from different classes, such as fluoroquinolones, beta-lactams, tetracyclines, aminoglycosides, and chloramphenicol among the thermophilic *Campylobacter* species (Hasdemir, 2007; Bardoň et al., 2017; Wysok et al., 2020). This resistance, which can occur in dual or more combinations and in different antibiotic groups, varies between 28% and 90.7% (Bardoň et al., 2017; Wysok et al., 2020). In this study, multiple antimicrobial resistance was found to be 57.6% for *C. jejuni* and 50% for *C. coli*, within the same rates and profiles reported in other studies. It is thought that the proportional differences may be caused by the type of sample taken, the sample area and the diversity of the antimicrobials used.

 In conclusion, phenotypic methods, PCR and MALDI-TOF MS were used together to identify thermophilic *Campylobacter* species and diagnostic values were found to be quite compatible with each other. It is thought that the MALDI-TOF MS, which offers a high (99.9%) confidence interval and high sensitivity in a few hours, may be a technique that can be applied in the diagnosis of pathogens in the field of veterinary science. Azithromycin and gentamicin resistance was found to be low among the thermophilic *Campylobacter* isolates, while resistance to others was quite high. Identification of genes responsible for the resistance to related antibiotics is important in terms of revealing the epidemiology of resistance, developing appropriate treatment strategies, and planning preventive measures correctly.

## Conflict of interest

 The authors declare that they have no conflict of interest.
